# The effect of *Aloe ferox *Mill. in the treatment of loperamide-induced constipation in Wistar rats

**DOI:** 10.1186/1471-230X-10-95

**Published:** 2010-08-19

**Authors:** Olubunmi A Wintola, Taofik O Sunmonu, Anthony J Afolayan

**Affiliations:** 1 Department of Botany, University of Fort Hare, Alice 5700, South Africa

## Abstract

**Background:**

Constipation is the most common gastrointestinal complaint all over the world and it is a risk factor of colorectal cancer. In this study, the efficacy of aqueous leaf extract of *Aloe ferox *Mill. was studied against loperamide-induced constipation in Wistar rats.

**Methods:**

Constipation was induced by oral administration of loperamide (3 mg/kg body weight) while the control rats received normal saline. The constipated rats were treated with 50, 100 and 200 mg/kg body weight/day of the extract for 7 days during which the feeding characteristics, body weight, fecal properties and gastrointestinal transit ratio were monitored.

**Results:**

The extract improved intestinal motility, increased fecal volume and normalized body weight in the constipated rats, which are indications of laxative property of the herb with the 200 mg/kg body weight of the extract showing the best efficacy.

**Conclusion:**

The effect of the extract compares favourably well with senokot, a standard laxative drug. These findings have therefore, lent scientific credence to the folkloric use of the herb as a laxative agent by the people of the Eastern Cape of South Africa.

## Background

Constipation is a highly prevalent functional gastrointestinal disorder affecting 3-15% of the general population [1,2]. In South Africa, 29% of the population, consisting of both black and white suffer from constipation especially in the elderly [3]. The menace has a substantial impact on morbidity and quality of life [4], which may be characterized by unexplained abdominal pain, discomfort and bloating in association with altered bowel habits [5].

The use of chemical drugs such as senna, correctol, exlax, senokot and gaviscon is very common as a means of treating constipation. Statistics have shown that 43% of whites and 76.6% of blacks in South Africa indulge in the use of laxatives, out of which 14.3% and 21.5% respectively use more than one laxative at a time for the treatment of constipation [3]. The use of these orthodox drugs is however, limited due to their high cost and undesirable side affects [6]. Consequently, majority of the affected persons in South Africa rely on herbal preparations for the treatment of the menace. For instance, some plant extracts are known to exhibit antispasmodic effects by stimulating water absorption in the intestine [7]. Apart from being fast acting, cheap and readily available, the users of medicinal plants for the treatment of constipation also believe that they have some control in their choice of medication [8].

*Aloe ferox *Mill. belongs to the family Asphodelaceae. The plant is widely distributed in the Southern Cape, Eastern Cape, Southern parts of KwaZulu Natal, the Free State and Lesotho. It is an arborescent perennial shrub with a single stem of 2-3 m in height. The plant is crowned by a large rosette of numerous leaves which are glaucous and oval-lanceolate. It is one of the widely used medicinal plants in traditional medicine because of its healing properties against many ailments [9]. For example, extract from the plant has been reported to be effective in the treatment of tooth abscesses [10], sexually transmitted infections [11], wound healing [12], arthritis and rheumatism [13], conjunctivitis and eye ailment [14] and as insect repellant [15]. The herb is also used traditionally as laxative; however, there is little or no scientific report to substantiate this claim. The present study was therefore, designed to evaluate the laxative activity of the aqueous leaf extract of *Aloe ferox *on loperamide-induced constipated rats and the effect was compared with senokot, a standard laxative drug.

## Methods

### Drugs and chemicals

Loperamide hydrochloride, carmine and carboxymethylcellulose were procured from Sigma Chemical Co., St Louis, MO, USA while senokot was a product of Reckitt Benckiser Pharmaceutical (Pty) Ltd, South Africa. All other chemicals and reagents used were of analytical grade.

### Plant materials

Fresh mature whole leaves of *Aloe ferox *were collected in Ntselamanzi Area of Nkonkobe Municipality in the Eastern Cape Province of South Africa. The Plant was authenticated by Prof DS Grierson; a botanist in the Department of Botany, University of Fort Hare and a voucher specimen (Wintola Med.2009/01) was prepared and deposited in the University herbarium.

### Preparation of aqueous extract

The leaves of *A. ferox *were thoroughly washed with distilled water, cut into thin slices and dried in the oven at 50°C for 24 h. The dried leaves were grinded into powder and 100 g of the material was extracted by shaking for 24 h in 1000 ml of distilled water on an orbital shaker (SO1 orbital shaker, Stuart scientific, Stone U. K). The extract obtained was filtered through Whatman No 1 (70 mm) filter paper and Freeze dried (Vir Tis benchtop k, Vir Tis company Gardiner NY) to give a yield of 24.4 g. This was reconstituted in distilled water to give the required doses of 50, 100 and 200 mg/kg body weight for the experiment.

### Animal used

Male albino rats (*Rattus norvegicus*) of Wistar strain with a mean weight of 140 ± 3.67 g were obtained from the experimental animal house of the Agricultural and Rural Development Research Institute (ARDRI), University of Fort Hare, Alice. The animals were housed individually in clean metabolic cages placed in a well ventilated house with optimum condition (temperature 23 ± 1°C, photoperiod; 12 h natural light and 12 h dark; humidity; 45-50%). They were acclimatized to the animal house condition for 7 days during which they were allowed free access to commercial pelleted rat chow (Pioneer Food (Pty) Ltd, Huguenot, South Africa) and water. The cleaning of the cages was done on a daily basis. All animal treatments were in accordance with international ethical guidelines and the National Institute of Health guide concerning the care and use of laboratory animals. The study was carried out following the approval from the Ethical Committee of the University of Fort Hare on the use and care of animals.

### Induction of constipation in the rats

Constipation was induced in the animals by oral administration of 1 ml loperamide (3 mg/kg body weight in 0.9% sodium chloride for 3 days) [16], while the control rats were administered with the normal saline only. The Passage of reduced, hard and dry fecal pellets indicated constipation in the rats.

### Experimental design

The rats were grouped into six of four rats each. The animals in Group 1 (control) and Group 2 (constipated control) were administered with distilled water. Groups 3, 4 and 5 comprised constipated rats given 50, 100 and 200 mg/kg body weight/day of *A. ferox *extract respectively while Group 6 were constipated rats administered with senokot. The administration was done using metal oropharyngeal cannula. The water intake, feed intake and body weight gain of all the rats were recorded during experimental period and treatment continued for 7 days.

### Total number, dry weight and water content of fecal pellet

The excreted fecal pellets of individual rats were collected everyday at 09:00 h throughout the duration of the experiment. Total number, weight and water content of the pellets were determined. The water content was calculated as the difference between the wet and dry weights of the pellet.

### Gastrointestinal transit (GIT) ratio

GIT ratio was measured according to the method of Nakagura et al. [17]. On the 7th day, 1 ml of carmine (3 g suspended in 50 ml of 0.5% carboxymethylcellulose) was orally administered to the rats. One hour after administering the marker, the animals were sacrificed and the small intestines were quickly removed. The distance over which the carmine had travelled and the total length of the small intestine were measured. The GIT ratio was expressed as the percentage of the distance travelled by the carmine relative to the total length of the small intestine.

### Statistical analysis

Data were expressed as means ± SD of four replicates and were subjected to one way analysis of variance (ANOVA) followed by Duncan multiple range test to determine significant differences in all the parameters. Values were considered statistically significant at p < 0.05.

### Results

Loperamide significantly reduced the water intake, the number, water content and the weight of the fecal pellets (Table 1). This was an indication of the induction of constipation in the rats. However, there was no significant difference in feed intake between the control and the constipated animals.

**Table 1:** Effect of loperamide on feed intake, water intake and fecal properties of constipated rats

Parameters	Normal Control	Constipated rats
Feed intake	15.32 ± 1.12	17.05 ± 0.74 *
Water intake	19.00 ± 1.41	8.92 ± 0.68 **
Number of fecal pellet	64.67 ± 2.80	24.04 ± 0.94**
Water content of fecal pellet	1.52 ± 0.17	0.57 ± 0.10**
Weight of fecal pellet	6.38 ± 0.30	2.95 ± 0.09**

Data are mean ± SD values (n = 4); *not significantly different; ** significantly different from normal control at P < 0.05

While water consumption decreased in the untreated constipated rats, the administration of aqueous extract of *A. ferox *significantly increased the water intake in constipated rats (Table 2). Again, there was no significant difference in the feed intake of all the animals. Similarly, the extract significantly increased the number, water content and weight of fecal pellets in the constipated rats in a dosage-dependent manner. The body weights of the constipated animals were also normalized following the treatment with the extract.

**Table 2:** Effect of aqueous extract of *Aloe ferox *on feed and water intake, body weight gain and fecal properties of constipated rats

Parameters	Normal control	Constipated control	Constipated + *A. ferox *(mg/kg body weight)			Senokot
			**50**	**100**	**200**	
Feed intake	17.18 ± 1.36^a^	19.23 ± 3.86^a^	19.90 ± 1.61^a^	20.54 ± 1.38^a^	17.80 ± 1.60^a^	19.97 ± 3.31^a^
Water intake	19.62 ± 2.22^a^	11.72 ± 2.47^b^	16.57 ± 2.05^a^	17.24 ± 0.17^a^	19.79 ± 2.33^a^	18.14 ± 0.61^a^
Number of fecal Pellet	73.57 ± 4.39^a^	38.20 ± 2.21^b^	45.43 ± 1.90^c^	57.57 ± 1.62^d^	69.83 ± 4.49^a^	63.00 ± 3.11^a^
Water content of fecal pellet (ml)	1.40 ± 0.08^a^	1.04 ± 0.09^b^	1.75 ± 0.21^c^	1.95 ± 0.11^c^	2.25 ± 0.21^d^	2.09 ± 0.06^d^
Weight of fecal pellet (g)	7.14 ± 0.23^a^	3.34 ± 0.38^b^	5.72 ± 0.18^c^	7.42 ± 0.33^a^	8.10 ± 0.72^a^	7.31 ± 0.25^a^
Body weight gain (g)	15.30 ± 1.00^a^	33.80 ± 1.00^b^	14.20 ± 0.71^a^	13.20 ± 2.16^a^	12.50 ± 1.85^a^	15.35 ± 1.21^a^

Data are mean ± SD values (n = 4). Row values with different superscripts from the control are significantly different (P < 0.05)

**Figure 1:**
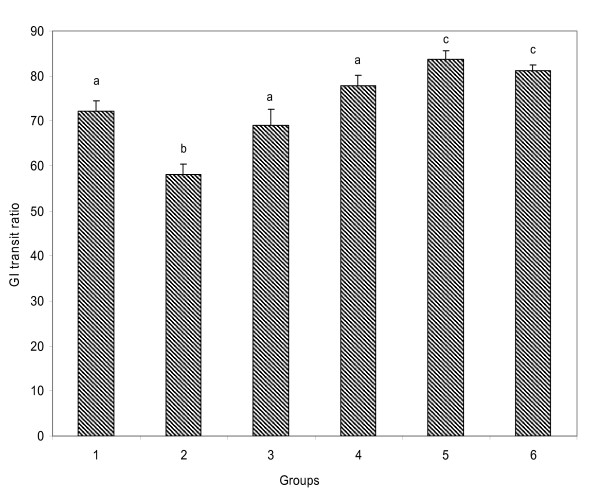
**Effect of aqueous extract of *Aloe ferox *on gastrointestinal transit ratio in loperamide-induced constipated rats**. Data are means of four determinations ± SD. Bars with different letters from the control are significantly different (P < 0.05).

Loperamide administration significantly reduced gastrointestinal motility in the untreated constipated rats (Fig.1). The treatment with the extract, however, increased the gastrointestinal movement in a dose dependent manner which compared favourably well with senokot, a standard constipation drug.

## Discussion

The use of herbal remedies in the treatment of constipation is a common practice in many countries of the world including South Africa [13,18]. The present study has clearly demonstrated that aqueous extract of *Aloe ferox *has laxative activity; which is comparable to senokot, a standard laxative drug.

The use of loperamide as constipation inducer is well documented. The drug inhibits intestinal water secretion [19] and colonic peristalsis [20]. This inhibition extends fecal evacuation time and delays intestinal luminal transit [21]. Loperamide-induced constipation is therefore considered to be a model of spastic constipation [22].

The observed reduction in the number, weight and water content of fecal pellets following the treatment with the drug indicated induction of constipation in the rats. Similar observation was reported by Shimotoyodome et al. [23]. The reduction in the water consumed by the constipated animals may also be due to the effect of the drug which probably accounted for the reduction in water content of the fecal pellets. However, the drug did not prevent the animals from feeding adequately.

The administration of aqueous extract of *A. ferox *to the constipated rats was effective in influencing increased defecation frequency, fecal volume and motility of the colon. These are indications of the laxative property of the plant extract. This may be due to the presence of anthranoid glycosides derivatives of which aloin is the main compound [24,25]. According to Izzo et al. [26], aloin is metabolized by the colonic flora to reactive aloe-emodin which is responsible for the purgative activity. This compound possibly exerts its action by disturbing the equilibrium between the absorption of water from the intestinal lumen via an active sodium transport [27] and the secretion of water into the lumen by prostaglandin-dependent mechanism [28,29].

Although the feed intake did not differ among the groups, the gain in body weight was higher in the untreated constipated rats compared to the extract treated groups. This may be due to the accumulation of fecal pellets in their bodies, thus accounting for the extra weight. This clearly indicates that the plant extract increased intestinal secretion and motility in the constipated rats. Similar observation was reported by Niwa et al. [30] where dietary fiber was used for the treatment of morphine-induced constipation in rats. Of particular interest is the fact that the effect of the extract of *A. ferox *was dose dependent in this study. The effect of the highest dosage actually compared favourably well with senokot.

The transit process of the entire gastrointestinal tract reflected the overall gastrointestinal motor activity. Measuring colonic transit time is useful in constipation, abdominal bloating and refractory irritable bowel syndrome. It also provides quantitative information about colonic transit, enables the identification and characterization of transit abnormalities and allows assessment of the severity of the problem as well as the response to therapy [31]. In this study, carmine was used as the marker used to measure the colonic movement. The extract increased intestinal motility which, in turn, enhanced colonic peristalsis in the rats. The possible mechanism of the extract in this process may be by enhancing the release of fluid thereby increasing intestinal secretion. The laxative effect of the extract could also be attributed to changes in the intestinal motility, which produced an increase in intestinal transit and colonic movement [32]. Generally, the effect of the treatment with the extract compared favourably well to that of senokot. This is an indication that the herb was effective in ameliorating bowel obstruction, thereby enhancing easy movement in the intestine.

## Conclusion

The present study revealed that oral administration of aqueous extract of *Aloe ferox *exhibited laxative activity in loperamide induced constipated rats. This suggests the beneficial effects of the herb in improving intestinal motility. It is, however, very important to note that the extract at 100 and 200 mg/kg body weight showed better laxative action than at 50 mg/kg body weight. The effect of the extract compared favourably with senokot. These findings have lent scientific support to the folkloric use of *A. ferox *as a laxative agent.

## Competing interests

The authors declare that they have no competing interests.

## Authors' contributions

OA participated in the design of the study, prepared the aqueous extract and carried out the study involving fecal pellets and gastrointestinal transit ratio. TO conceived of the study, participated in its design and performed the statistical analysis. AJ participated in the design of the study and coordination and helped to revise the manuscript. All authors read and approved the final manuscript.

## Pre-publication history

The pre-publication history for this paper can be accessed here:

http://www.biomedcentral.com/1471-230X/10/95/prepub
